# SCO-Spondin Derived Peptide NX210 Induces Neuroprotection *In Vitro* and Promotes Fiber Regrowth and Functional Recovery after Spinal Cord Injury

**DOI:** 10.1371/journal.pone.0093179

**Published:** 2014-03-25

**Authors:** Laurent Sakka, Nathalie Delétage, Fabrice Lalloué, Amélie Duval, Jean Chazal, Jean-Jacques Lemaire, Annie Meiniel, Hubert Monnerie, Stéphane Gobron

**Affiliations:** 1 Image-Guided Clinical Neuroscience and Connectomics, EA7282, Faculté de Médecine, Université d'Auvergne, Clermont-Ferrand, France; 2 Neuronax, Biopôle Clermont-Limagne, Saint-Beauzire, France; 3 Service de Neurochirurgie, Centre Hospitalier et Universitaire de Clermont-Ferrand, Clermont-Ferrand, France; 4 Homéostasie Cellulaire et Pathologies, EA3842, Faculté de Médecine, Limoges, France; 5 Laboratoire GReD/Inserm U 110/CNRS, UMR 629, Faculté de Médecine, Université d'Auvergne, Clermont-Ferrand, France; 6 Department of Neurology, Children's Hospital of Philadelphia, Philadelphia, Pennsylvania, United States of America; Temple University School of Medicine, United States of America

## Abstract

In mammals, the limited regenerating potential of the central nervous system (CNS) in adults contrasts with the plasticity of the embryonic and perinatal periods. SCO (subcommissural organ)-spondin is a protein secreted early by the developing central nervous system, potentially involved in the development of commissural fibers. SCO-spondin stimulates neuronal differentiation and neurite growth *in vitro*. NX210 oligopeptide was designed from SCO-spondin's specific thrombospondin type 1 repeat (TSR) sequences that support the main neurogenic properties of the molecule. The objective of this work was to assess the neuroprotective and neuroregenerative properties of NX210 *in vitro* and *in vivo* for the treatment of spinal cord injury (SCI). *In vitro* studies were carried out on the B104 neuroblastoma cell line demonstrating neuroprotection by the resistance to oxidative damage using hydrogen peroxide and the measure of cell viability by metabolic activity. *In vivo* studies were performed in two rat models of SCI: (1) a model of aspiration of dorsal funiculi followed by the insertion of a collagen tube *in situ* to limit collateral sprouting; white matter regeneration was assessed using neurofilament immunostaining; (2) a rat spinal cord contusion model to assess functional recovery using BBB scale and reflex testing. We demonstrate for the first time that NX210 (a) provides neuroprotection to oxidative stress in the B104 neuroblastoma cells, (b) stimulates axonal regrowth in longitudinally oriented neofibers in the aspiration model of SCI and (c) significantly improves functional recovery in the contusive model of SCI.

## Introduction

The potential of the human central nervous system (CNS) to regenerate is strictly limited in adults and recent clinical studies have demonstrated a mixed effectiveness of medical treatments in significantly improving the neurological outcome of spinal cord-injured patients. To develop efficient treatments, it appears mandatory to consider the different events that take place in the two phases currently described in the pathophysiology of the traumatic process. In the primary phase, mechanical factors such as the displacement of vertebral column components, lead to a distraction, a compression, or a section of the spinal cord resulting in initial tissue destruction. Around the primary lesion made of axonal and vessel disruptions, myelin sheath alterations, neuronal and glial cell death, a peripheral rim of spared tissue connects both stumps of the spinal cord. The aim of early surgical management is to alleviate the mechanical insult to the spinal cord if only to preserve these non-sectioned axons that cross the injury site. The rational of this procedure has been proven to be efficient in the management of acute SCI if performed in the first hours following the trauma [1], [2]. In the secondary injury phase, vascular disorders, ischemia, inflammation, glutamate-mediated excitotoxicity and Ca^2+^ dyshomeostasis result in the extension of the primary tissue damage [3]. To counteract the consequences of inflammation and excitotoxicity, methylprednisolone has been tested in an extensive double-blind randomized trial that demonstrated a beneficial effect when administered within 8 hours following injury [4], [5]. This has led to its prescription as a neuroprotectant in the early medical management of acute SCI, but its widespread use remains debated by some institutions [6]. Free radicals, and particularly reactive oxygen species, have been demonstrated to induce lipid peroxidation, cell membrane damage and ionic homeostasis disruption that propagate neuronal and glial cell death beyond the initial site of injury [7]. The secondary injury phase is considered a possible target of therapeutic approaches using neuroprotective agents and particularly antioxidant molecules. Unfortunately, most of these molecules have failed to transform the clinical outcome of patients after SCI. Their mixed efficiency can be explained by the fact that functional recovery is also limited by the poor regenerating capacity of the adult central nervous system.

In this context, tremendous progress has been made over the past three decades in understanding the biological mechanisms that inhibit CNS regeneration. Thus, an extensive number of pharmacological agents has been investigated to counteract these inhibitory processes or to support neural circuitry reconstruction. There is evidence that blocking individual inhibitors of white matter regeneration or inhibitory pathways by these pharmacological agents lead to the modulation of other inhibitory mechanisms left unimpaired. These compensatory events could help explain why functional results are so disappointing when moving from laboratory to clinical studies and underline the need for treatments acting through complementary pathways. To date, practitioners still lack therapeutic solutions in the management of SCI.

Interestingly, the limited regenerating potential of adult CNS contrasts with the amazing plasticity displayed by the embryonic and perinatal CNS [8], [9]. This might be due to the change in the expression of extracellular matrix (ECM) components that promote neuronal circuit formation and plasticity during embryonic and postnatal development, but consolidate neuronal circuitry and inhibit regeneration in adults. Based on this observation, we focused on SCO-spondin, a large multi-domain protein of the ECM secreted by ependymal cells in early neurulation [10]. Widely distributed in the CNS, SCO-spondin may be involved in the development of brain commissural fibers and the regeneration of the spinal cord in vertebrates [11]–[14]. In the cerebrospinal fluid compartment of the CNS, SCO-spondin naturally aggregates into the Reissner's fiber, a thread-like structure present in the central canal of most vertebrates which stimulates neurite outgrowth *in vitro* [15]. Molecular characterization of this biogenic glycoprotein showed several thrombospondin type-1 repeats (TSR) consensus domains involved in cell-cell and cell-matrix interactions, neuronal differentiation and axonal pathfinding [16], [17]. Designed from SCO-spondin-specific TSR sequences, NX210 oligopeptide has been shown to provide the *in vitro* effects of the biomolecule [18], [19]. However, while we have shown the capacities of NX210 to increase cell survival and to induce neurite outgrowth, we have not investigated its *in vitro* effects on neuroprotection and its *in vivo* efficacy in axonal regrowth and functional recovery.

This paper presents the neuroprotective and neuroregenerative properties of an embryonic biomolecule-derived peptide: (1) neuroprotection was assessed with a test of resistance to H_2_O_2_ toxicity using a B104 neuroblastoma cell line; (2) axonal regrowth was explored in a rat model of SCI by dorsal hemi-section and insertion of a collagen tube, and (3) functional recovery was tested in a contusive model of SCI.

## Materials and Methods

### The pharmacological compound

NX210 was obtained by chemical synthesis (Polypeptides Laboratories, Strasbourg, France). The amino acid sequence –WSGWSSCSRSCG- has been previously detailed [17]. For each experiment, NX210 was extemporaneously suspended in sterile water before administration. For *in vivo* studies, NX210 was delivered at a dosage of 100 μg/kg.

### Neuroprotective effects of NX210 *in vitro*: resistance of a B104 neuroblastoma cell line to H_2_O_2_ toxicity

B104 neuroblastoma cells (HPA Culture Collections Salisbury, UK) were plated onto poly-D-lysine-coated 96-well plates at a final density of 20 000 cells/well. Cells were grown for 24 hours in Dulbecco's Modified Eagle's Medium (DMEM) (Lonza, Levallois, France) supplemented with 2 mM glutamine, 100 U/ml penicillin G, 100 μg/ml streptomycin sulfate and 10% fetal bovine serum (FBS) (Invitrogen, Carlsbad, USA). Five hours after cell seeding, the culture medium was replaced by 200 μl/well of medium without FBS. After 24 hours of serum deprivation, cells were treated for 18 hours with 100 μl/well of 0.15 mM H_2_O_2_ (Sigma, Saint-Quentin-Fallavier, France) in the presence of NX210 (100, 250, 500 μg/ml) or the vehicle. The concentration of H_2_O_2_ was determined according to preliminary experiments. Dilutions of H_2_O_2_ were made from a 30% solution into DMEM just prior to each experiment. Cell viability in response to the different treatments was evaluated using a WST-1 assay (Roche, Switzerland). Triplicate wells were used for each condition and each experiment was repeated four times. Briefly, 10 μl/well of WST-1 reagent was added and B104 cells were incubated for 1 hour at 37°C and 5% CO_2_.The absorbance of the samples was measured at 450 nm using a microplate reader (Multiskan Spectrum, Thermo Fisher Scientific, USA) with a background control as the blank. The cell survival ratio was expressed as the percentage of the untreated control. Morphological changes were observed using an inverted microscope (Eclipse TS100, Nikon, Japan) and a digital camera (DS-Vi1, Nikon, Japan).

### *In vivo* study of NX210 in rat SCI

#### Ethics Statement

*In vivo* procedures were performed in compliance with the EU Directive 2010/63/EU on the protection of animals used for scientific purposes and the NIH guidelines for the care and use of laboratory animals. Animal experiments were approved by the Ethical Committee of the National Laboratory Animal Center, Kuopio, Finland (Permit Number: ISLH-2006-02494/Ym-23).

#### Animals

Procedures were performed on adult female Sprague-Dawley rats (240–300 g). Surgical procedures were carried out under general anesthesia using 5 mg/kg xylazine i.p. and 75 mg/kg of ketamine i.p. Animals were housed in individual standardized cages at 22±1°C in a light-controlled environment (12-hour-light), and given food and water *ad libitum*. Animals were clinically observed twice a day all along the study and welfare-related assessment was carried out before each testing. The administration of analgesic drug was not necessary because animals didn't show any sign of pain or severe distress.

#### Animal models of SCI and surgical procedures

##### Effects of NX210 on neurite regrowth in vivo

Ten animals were used. After T9-T10 laminectomy and midline dorsal incision of the dura mater, gentle aspiration of both posterior white columns (dorsal funiculi) and both dorsal horns of the spinal cord with a fine-glass micropipette under the operating microscope created a cavity of 5 mm long and 1 mm deep [20]. A placental type-IV collagen tube (Imedex, Saduc, France) filled with 10 μl of saline (control, n = 5) or a NX210 solution at 2.5 μg/μl (n = 5) was placed into the cavity. The dura mater and the skin were sutured. The animals were euthanized at day 10 (D10) with 80 mg/kg pentobarbital i.p., before intracardiac injection of heparinized (2.5 IU/ml) serum and intracardiac perfusion with 4% paraformaldehyde.

Blocks containing the lesion were excised, post-fixed in 4% paraformaldehyde 24 hours at 4°C and transferred into a 30% sucrose solution in PBS. The samples were frozen at −50°C in isopentane and embedded in OCT compound (Tissue-Tek, Elkhart, USA). Cryostat sagittal sections of 16 μm thick were fixed on glass slides precoated with aminopropyltriethoxysilane (Fluka, Buchs, Switzerland). Sections were incubated overnight at RT with primary mouse antibodies against neurofilament (NF) (Dako, Glostrup, Denmark) diluted 1∶200 and primary rabbit antibodies against laminin (Abcam, Paris, France) diluted 1∶100 in 0.01 M PBS containing 1% Triton X-100 and 10% normal goat serum (NGS). The next day, sections were washed and incubated 2 hours with Cy-3 conjugated goat anti-mouse antibodies diluted 1∶400 and FITC conjugated goat anti-rabbit antibodies (Interchim, Montluçon, France) diluted 1∶200 both in 0.01 M PBS containing 1% Triton X-100 and 10% NGS. Sections not incubated with primary antibodies were used as control to detect nonspecific binding. The slides were observed at a 72X magnification and digitized to measure the extent of fiber regrowth with ImageJ software, 1.43u (NIH, USA).

##### Effects of NX210 on functional recovery

Sixteen animals were used. After T9-T10 laminectomy, SCI was performed by dropping a 10 g rod (2.5 mm diameter) from 12.5 mm upon the exposed dura mater using a NYU/MASCIS Impactor. The meningeal sheath was punctured with a 30-gauge needle. Five minutes after the injury, 100 μg/kg NX210 (n = 8) or the vehicle (n = 8) was randomly administered with a 10 μl Hamilton syringe through the site of puncture into the lesion. The volume (3 μl) was injected over 1 minute. The wound was sutured and the rats were housed in individual cages. Two days later, the injection was repeated. Manual bladder expression was performed twice a day until bladder emptying reflex recovery.

Behavioral testing was performed with a standard square open-field arena divided into 16 cells. Animals were observed individually for 10 min by two blinded observers. The path length and the percentage of time spent in central cells during this time of observation were noted, and the results were expressed as a ratio of the animals' performance in comparison to baseline data (set at 100%).

Locomotor performance was assessed by two blinded observers with the Basso, Beattie, Bresnahan Locomotor Rating Scale (BBB scale) [21], [22]. Each assessment was realized before SCI procedure (baseline) and once a week thereafter. Ambulation was recorded by the TrueScan Photo Beam Activity system (Coulbourn Instruments) for 10 minutes. Body weight (BW) was assessed before and once a week after surgery.

Reflex testing was performed by two blinded observers before and once a week after injury. The reflexes chosen to supplement the assessment of functional recovery were toe spread and paw placement. The toe spread reflex was studied by lifting the rat by its tail with legs hanging free and observing the spread of the toes. The placing reflex was studied by lifting the rat by the trunk with legs hanging free. The dorsal and lateral aspects of each foot were rubbed against the table edge and the speed and accuracy in placing the foot on the table were evaluated [23]–[25]. A 4-point scoring system was used: 0: no reflex, 1: considerably weaker than normal, 2: slightly weaker than normal, 3: normal.

The animals were euthanized at day 30 (D30) with 80 mg/kg pentobarbital i.p., before intracardiac injection of heparinized (2.5 IU/ml) serum and intracardiac perfusion with 4% paraformaldehyde.

### Quantification and statistical analysis

Data were expressed as the mean ± SEM. For *in vitro* studies, comparison between NX210-treated, vehicle-treated groups and control was made using one-way ANOVA followed by a post-hoc test of Dunnett. For *in vivo* studies, ANOVA for repeated measures and non-parametric Friedman test followed by post-hoc test of Tukey-Kramer were used to study the differences in parameter evolution between groups. These analyses were complemented with random-effect models that measure the within-subject correlation by taking into account fixed effects (time, group and interaction) and random effects (random intercept and random slope models). Statistical tests were performed for a Type I error α = 5%. The statistical analyses of *in vitro* and *in vitro* studies were performed using STATAv10 software (StataCorp, College Station, Texas, USA).

## Results

### Neuroprotective effects of NX210 *in vitro*

**Figure 1:**
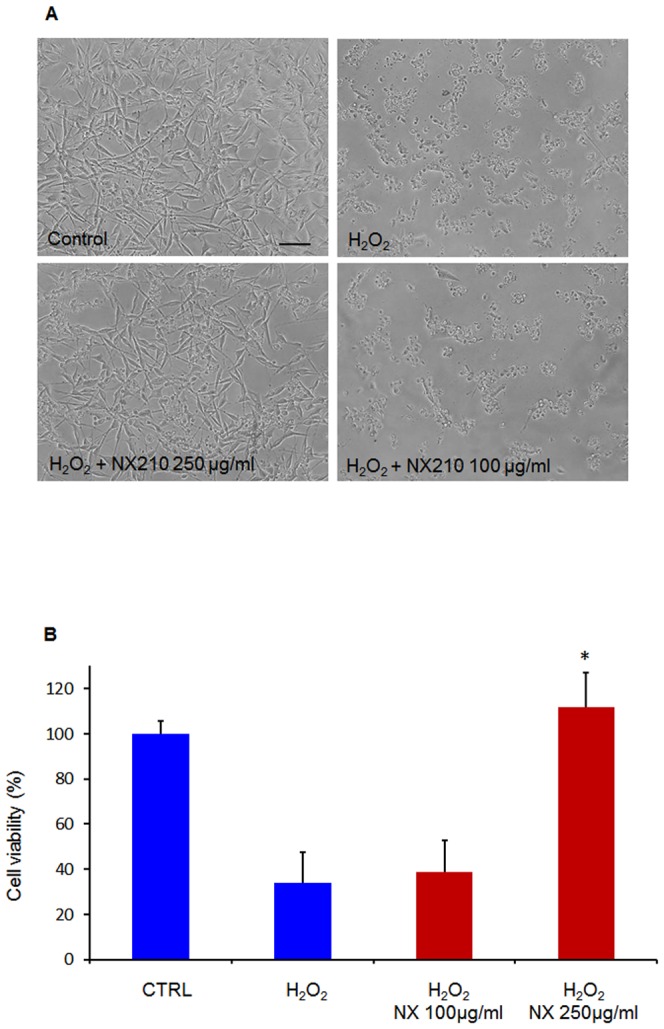
Neuroprotective effect of NX210 against H_2_O_2_ using a B104 cell line. Neuroprotective effect of NX210 at 100 and 250 μg/ml was assessed by the resistance of a B104 neuroblastoma cell line after 18 hours of exposure to H_2_O_2_. (A) Morphological observation using phase-contrast microscopy shows normal features of B104 cells in the control group. Exposure to H_2_O_2_ alone decreases B104 cell viability, normal B104 cells are partly replaced by numerous cellular debris. Treatment with NX210 250 μg/ml significantly prevents H_2_O_2_-induced cell death. (B) Viability of B104 cells treated with NX210 was assessed using the WST-1 assay. H_2_O_2_-induced cell death was significantly prevented in NX210-treated cultures at the concentration of 250 μg/ml. Each experiment was performed in triplicate and repeated 4 times. Data represent the percentage of live cells relative to controls (no treatment) and are presented as mean values +/- SEM. * significantly different from H_2_O_2_-treated cells, p<0.001. Scale bar  =  100 μm.

The neuroprotection provided by NX210 was assessed by measuring the resistance of B104 neuroblastoma cells to H_2_O_2_. For that purpose, we used the WST-1 assay, a colorimetric test designed to measure cell viability by extracellular enzymatic reduction of water soluble tetrazolium (WST) into formazan. H_2_O_2_-induced B104 cell death was apparent after 18 hours in culture, with a 70% reduction in cell viability compared to controls. In sharp contrast, NX210 at 250 μg/ml prevented the effect of H_2_O_2_ on B104 cells (Fig. 1). These data suggest a neuroprotective effect of NX210 against oxidative damage mediated by reactive oxygen species.

### Effects of NX210 on neurite regrowth *in vivo*

**Figure 2:**
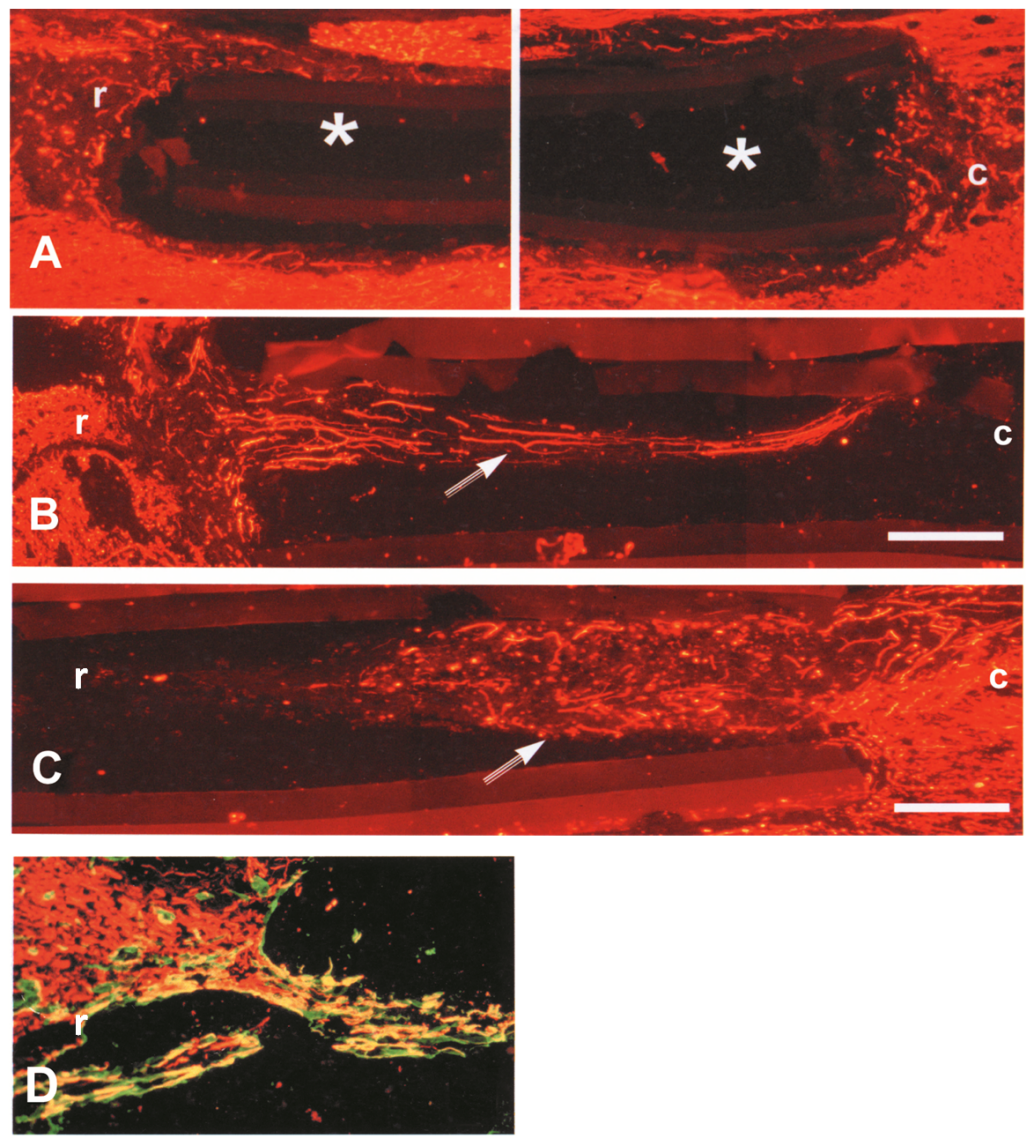
*In vivo* effects of NX210 on neuritic regrowth in a rat model of SCI. The injury was performed by aspirating the dorsal funiculi and the dorsal horns of the spinal gray matter. A collagen tube filled with the vehicle (A) or NX210 (B and C) was placed into the site of injury. Neuritic regrowth was detected using neurofilament immunostaining. (A) In the vehicle group (n = 5), the collagen tube remains empty without neuritic regrowth (*). (B and C): in the NX210 group (n = 5), neuritic regrowth (arrow) is clearly observed inside the collagen tube, mostly from the caudal end of the lesion. Neofibers display a longitudinal arrangement. (D): At the surface of the growing processes, NF was co-localized with laminin, a marker of the basal lamina. r: rostral; c: caudal; Scale bar: 250 μm.

The collagen channel inserted into the injury site was well-tolerated by the spinal cord without major inflammatory reaction. In 4 out of the 5 control animals, neither axonal regrowth nor connective tissue was observed in the collagen tube (Fig. 2A). Only in 1 animal of this group, a limited neo-tissue formed from the caudal stump of the spinal cord was observed that stained positive for NF and not arranged in fibers. In sharp contrast, 4 out of the 5 NX210-treated animals showed patent fiber regrowth inside the collagen tube. In these animals, a strong positivity for NF labeling was observed in longitudinally oriented processes. Overall, fiber regrowth was more patent from the caudal stump compared to the rostral stump of the spinal cord in terms of length and diameter of the growing processes. Within 10 days following injury, neuritic fibers could grow up to 1.8 mm and 1.6 mm from the caudal and the rostral stumps, respectively (Fig 2B,2C). At the surface of the growing processes, NF was co-localized with laminin, a marker of the basal lamina (Fig. 2D). One animal from the treated group showed fiber regrowth only from the rostral end of the cavity.

### Effects of NX210 on functional recovery

**Figure 3:**
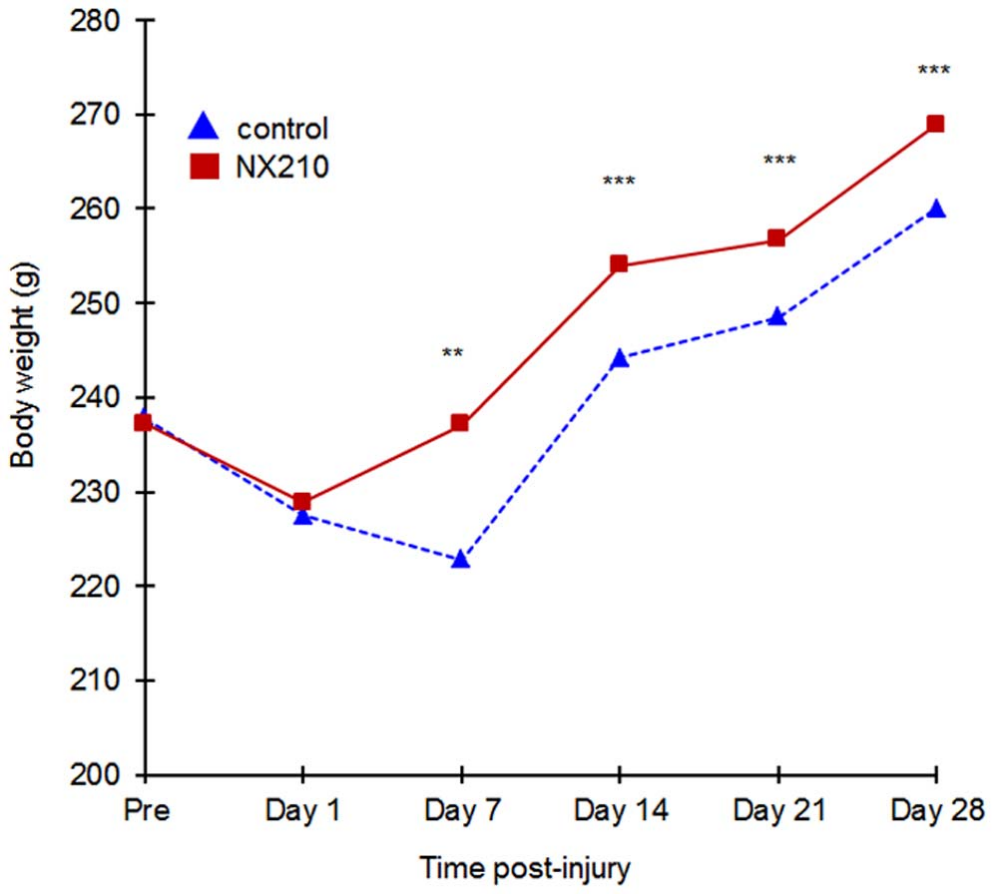
Effects of NX210 on functional recovery after SCI: body weight. A contusion was performed with the NYU/MASCIC impactor. The body weight was measured once a week over the month of the study. NX210-treated group (n = 8) in solid red line, vehicle group (n = 8) in blue dashed line. NX210 treatment induces a significant increase in body weight from D1, reaches the pre-injury value at D7 and keeps increasing until the end of the study. In sharp contrast, the body weight of the control group decreases in the first week, starts to increase at D7, reaches the pre-injury value at D14 but remains inferior to the body weight of the treated group throughout the study. Data are presented as mean ± SEM. Significantly different from baseline value, ** p<0.05 and *** p<0.001.

Before injury, there was no difference between NX210 and vehicle groups in term of BW, which decreased similarly in all groups after injury. Two animals from the control group died at D2 and D5. The BW of the NX210-treated group increased from D1 until the end of the study (D28) compared to the vehicle group, and reached presurgical values at D7. In contrast, the BW of the vehicle group reached presurgical values only at D14 (Fig. 3).

**Figure 4:**
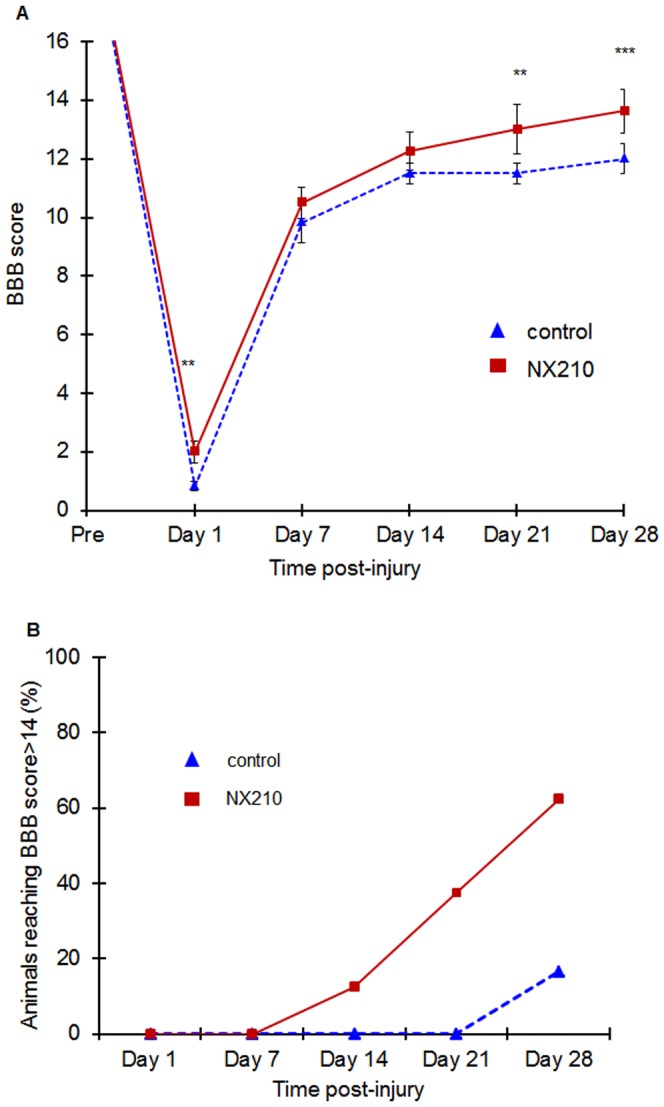
Effects of NX210 on functional recovery after SCI: BBB score. Functional recovery was assessed once a week over the month of the study using the open-field locomotion test and the BBB score. NX210-treated group (n = 8) in solid red line, vehicle group (n = 8) in blue dashed line. (A) The NX210-treated group BBB score was significantly higher in the last two weeks of observation compared to the vehicle group. BBB score continues to improve at the end of the study whereas in the control group, the BBB does not significantly improve from D14 to the end of the study. (B) The percentage of animals reaching a score superior to 14, corresponds to a complete forelimb – hind limb coordination. In the NX210-treated group, the animals reached a score >14 as early as the second week post-injury. The number of animals reaching a BBB score of 14 continuously increases until the end of the study reaching 62% of the NX210-treated group. In the vehicle-treated group, a score>14 is reached by only one animal at the fourth week post-injury. Data are presented as mean ± SEM, p<0.05.

The BBB score decreased significantly at all test-points after SCI, compared to the baseline score for all the animals. However, the NX210-treated group had significantly higher BBB score than the vehicle group from D14 to the end of the study (Fig. 4A). Also, the NX210-treated group's score kept increasing throughout the study whereas the control group seemed to reach a plateau at D14. Furthermore, the final BBB score of 14, which corresponds to a consistent coordination between forelimbs and hind limbs, was reached by 5/8 animals from the NX210-treated group as opposed to only 1/6 animals from the vehicle group (Fig. 4B).

**Figure 5:**
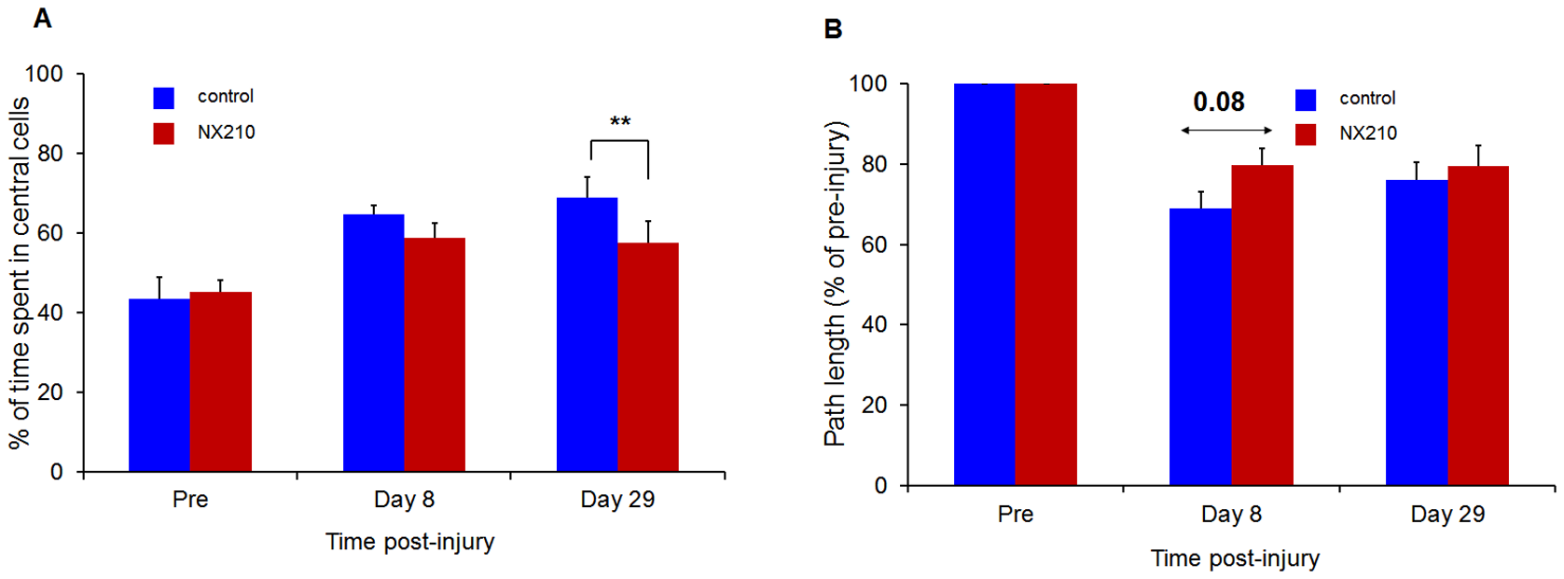
Effects of NX210 on functional recovery after SCI: open arena test. (A) Percentage of time spent in central cells. (B) Path length. NX210-treated rats (n = 8, red) showed significant improvement in the percentage of time spent in central cells and path length at post-injury time points compared to vehicle-treated rats (n = 8, blue) and the difference between treated and vehicle groups increased throughout the study for the percentage of time spent in central cells. Data are presented as group mean ± SEM, * p<0.05.

Compared to pre-injury values, the vehicle group showed significant impairment in the open arena tests at all post-injury time-points (2-way ANOVA, p<0.05): path length and percentage of time spent in central cells. In contrast, the NX210-treated group showed a slight improvement of path length values at intermediary test-points and a significant increase in the percentage of time spent in central cells, particularly at the end of the study (Fig. 5).

**Figure 6:**
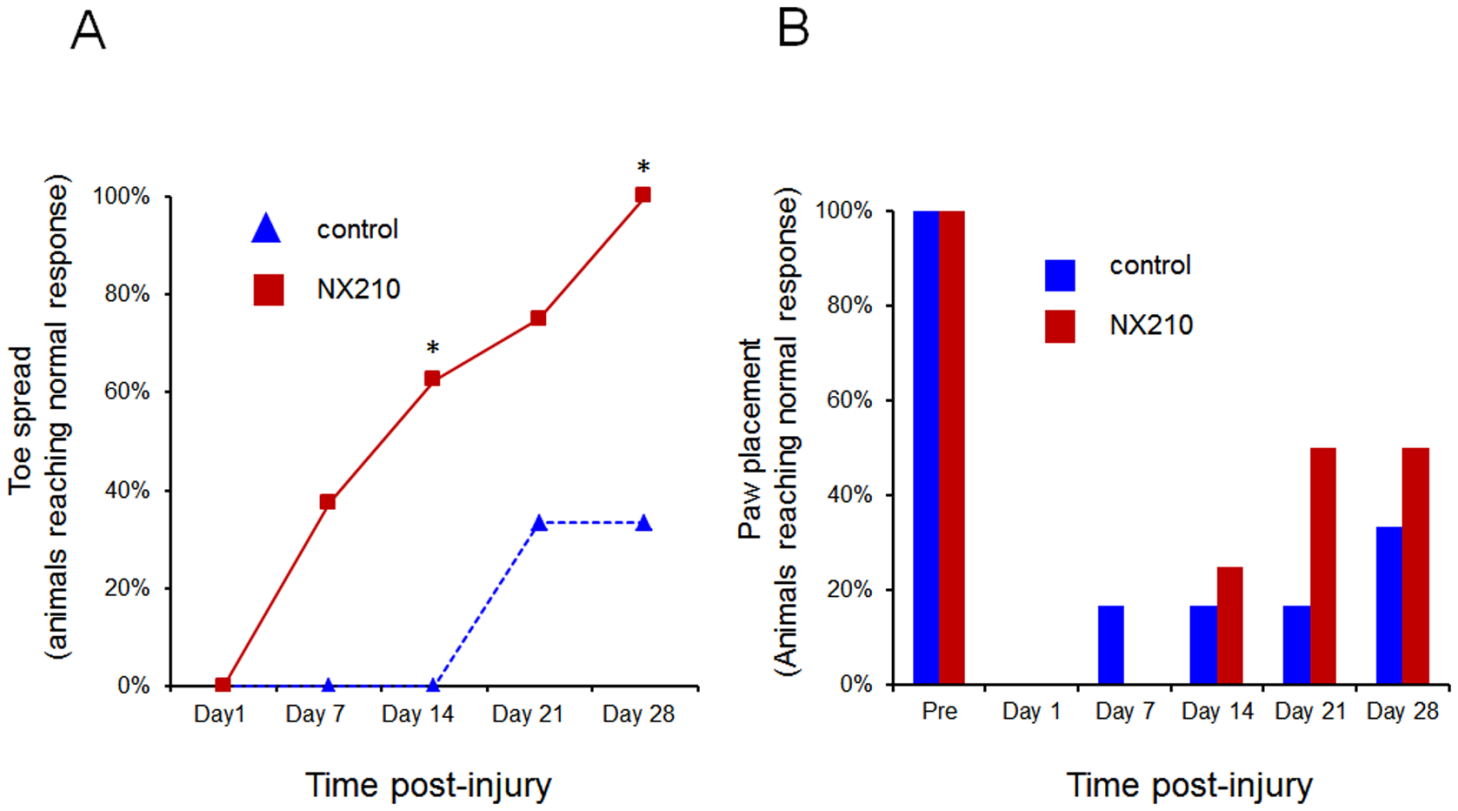
Effects of NX210 on functional recovery after SCI: reflex testing. Data are expressed as the percentage of animals providing a normal response to the tests (A) Toe spread. (B) Paw placement. Compared to pre-injury values, NX210-treated rats (n = 8) and vehicle-treated rats (n = 8) showed a significant impairment in both tests the day following the injury. However, at D21 and D28 post-injury, the NX210-treated group showed a significant improvement. Considering toe spread reflex, animals of the vehicle group reach a plateau at D21 whereas in the NX210 treated group, all the animals recover at the end of the study. Data are presented as group mean ± SEM. * significantly different from vehicle group, p<0.05.

Toe spread and paw placement scores were expressed as a percentage of animals reaching normal response. They were consistently higher in the NX210-treated group than in the vehicle group at D14, D21 and D28. Furthermore, in the NX210-treated group, 40% of the animals recovered a normal response to toe-spread reflex at D7, and this percentage kept increasing at all test-points thereafter, reaching 100% at the end of the study. Conversely, in the vehicle group, 33% of the animals recovered a normal response to toe-spread reflex, but only at the last two test-points (D21 and D28), and none of the animals reached 100%, remaining below the 40% mark. Thus, all the animals which received NX210 treatment reached a normal toe spread response by the end of the study. The responses to paw placement reflex tended to show an improvement following NX210 treatment since 50% of the NX210-treated animals recovered a normal response to paw placement at D21 and D28 versus 33% in the vehicle group (Fig. 6).

## Discussion

Restoring the functional connectome after a spinal cord injury should ideally combine neuroprotective and neuroregenerative treatments. Following the primary damage, free radicals largely contribute to the secondary injury mechanisms. Particularly, reactive O_2_ species (ROS) induce membrane lipid peroxidation resulting in the disruption of ionic homeostasis, the alteration of action potential propagation and cell death. Experimental data show that ROS concentration increases in the first 12 hours following the injury, and remains stable one week before recovering its basal value in the next 4 weeks [26]. Hydrogen peroxide is considered a prominent contributor to oxidative damage and is commonly used as a reference molecule to assess potential antioxidant agents *in vitro*. The significant reduction of H_2_O_2_-induced cell death obtained by the treatment with NX210 in a B104 cell line suggests a promising anti-oxidative property of the molecule. It is interesting to correlate these results to the decrease in free radical formation obtained by an integrin inhibitor [27], since most of NX210's effects are mediated by β1 integrin receptors [28]. At the present day, the efficacy of neuroprotective therapeutics remains limited in clinical practice, because in case of extensive destruction of the spinal cord the potential targets for neuroprotection may not be sufficient to support functional restoration. In this context, it seems crucial to address the second aspect of clinical recovery, i.e. neuroregeneration, to compensate for the axonal and glial cell loss. Neuroregeneration requires proper axonogenesis, pathfinding, synaptogenesis and myelination. The present study demonstrates the efficacy of the SCO-spondin derived peptide NX210, in improving axonal regrowth and functional recovery in 2 adult animal models of SCI.

SCO-spondin is a member of the thrombospondin type-1 repeat superfamily. This glycoprotein is a component of the extracellular matrix secreted by the subcommissural organ, a specialized area of the rostral *aqueductus cerebri* ependyma, and by specific ependymal cells of the central canal which might play a crucial role in spinal cord regeneration [29]–[31]. In addition, its stimulating actions on axonogenesis and pathfinding demonstrated *in vitro* and suggested *in vivo* in the early development of the posterior commissure [14], [32] make SCO-spondin an important morphogen.

NX210 is a dodecapeptide derived from the most conserved sequence of SCO-spondin TSR domains. TSR are functionally important domains involved in various biological systems that mediate cell-cell and cell-matrix interactions [16]. Additionally, these TSR domains are known to be attractive cues for axon guidance [33]. NX210 properties on neuritogenesis and synaptogenesis were carried out in a B104 neuroblastoma cell line. Neuron-like B104 cells have been commonly used as a model to study neuronal processes at a cell and molecule levels [34], [35]: they generate action potentials [36], release neurotransmitters [37] and their being individually identifiable in culture makes possible quantified analyses [38]. In addition, they are suitable for the study of growth factors since their culture does not require the addition of serum or growth factors. We have previously demonstrated that NX210 promotes neurite extension and fasciculation in cultured spinal cord neurons [19] and neuritogenesis in B104 neuroblastoma cells [17].

Our model of SCI by aspiration of dorsal funiculi and insertion of a collagen channel was derived from the model described by Duchossoy et al, 2001 [20] and designed to favor fiber regrowth and limit collateral sprouting: (1) total aspiration of both dorsal white columns (dorsal funiculi) and both dorsal horns (grey matter) of the spinal cord created a lesion without a peripheral rim of spared white matter; (2) the collagen tube inserted into the site of injury restricted the regrowth to the fibers coming from both spinal stumps. Remarkably, fiber regrowth was observed early after injury (D10), as compared to previously published data [39], [40] and predominated at the caudal end. The regrowth was about 2 mm/10 days, which corresponds approximately to a rat spinal segment [41]. The growth of longitudinally oriented neofibers is consistent with the neurite fasciculation elicited by the biogenic substance *in vitro* [15] and *in vivo* [14]. Indeed, the involvement of an other member of the TSR superfamily, F-spondin, has been demonstrated to promote fasciculation in the constitution of commissural axons *in vivo* [42]. In addition, the fasciculation of neuronal extensions has been suggested to favor neurite growth in the non-permissive environment of neuroregeneration [43]. Interestingly, neurofilaments were co-localized with laminin in regenerating fibers of NX210-treated animals. Indeed, laminins are a major component of the basal lamina produced by astrocytes or Schwann cells, which supports neurite growth, axonal guidance and neuronal migration through an interaction with β1 integrins [44]–[46]. Moreover, the natural growth capacity of serotoninergic fibers has recently been shown to be partly supported by laminin [47].

The contusive model, more akin to the lesions observed in human SCI [48]–[50], was used to evaluate the clinical efficacy of the molecule. The BW which assesses the animal well-being and its motility to get food, increased in the NX210-treated group one week earlier than the vehicle group and remained significantly higher until the end of the study. The effects on locomotion were evaluated using the open-field activity test and the BBB scale.

NX210 induced a slight improvement in path length values at intermediary test-points and a significant decrease in the percentage of time spent in central cells particularly at the end of the study as compared with the vehicle. Open-field activity tests provide an evaluation of motor behavior and general health through the assessment of animals' exploratory behavior. Even if exploratory activity may be modified by anxiety, habituation and locomotor training, open-field activity tests are sensitive to detect individual differences in neurological status even in severely damaged animals [25], [49], [51]–[53].

NX210 induced a significant improvement of hind limb function as shown by the BBB scores. Six out of 8 animals recovered a consistent weight support and complete forelimbs-hind limbs coordination with a BBB score>14. The differences in BBB scores between groups may appear small since the BBB scale, like all locomotor scales, is non-linear. In addition, the BBB scale gives only a global assessment of spinal function, that's why the BBB scale was combined with reflex testing. Placing and toe-spread reflexes commonly disappear the days following SCI and provide an abnormal response before normalizing progressively [23], [54]. Toe-spread reflex has been correlated to the volume of the lesion [24] but the anatomical substrate of these reflexes is not clearly understood. Experimental data describe placing and toe-spread reflexes not to be spinal reflexes. Placing reflex has been described as being under the control of the sensorimotor cortex, the cerebellum and the red nucleus and therefore its restoration after SCI should require corticospinal tract and extero/proprioceptive afferences integrity [25], [49], [54]–[59]. Hence, higher test results achieved by NX210-treated animals could be partly correlated to a supraspinal control restoration. We cannot rule out the hypothesis that collateral sprouting partly supported the functional improvement demonstrated by a treatment with NX210 in our contusion model, since this type of model leaves a peripheral rim of spared white matter from which collateral sprouting might take part in the formation of intraspinal circuits. Collateral sprouting from lesioned hindlimb corticospinal tract axons has been shown to develop synapses with long propriospinal neurons that bridge the lesion, connect with lumbar motor neurons and create new spinal circuits [60]. Hemi-section models of SCI with further re-lesion experiments could complement our contusive model to ensure functional recovery is supported by the regeneration of axons more than collateral sprouting [61]. We also speculate that the functional recovery might be partly supported by the NX210's neuroprotective property demonstrated by our *in vitro* experiments using B104 resistance to H_2_O_2_.

Myelination appears as the challenge of any successful neuroregenerative treatments. Indeed, poorly myelinated axons resulting from demyelination of spared axons or limited myelination of growing axons, may account for partial recoveries after SCI [62], [63]. Recent data suggest a promising effect of NX210 on myelination: (1) NX210 enhances oligodendrocyte survival and promotes cytoplasmic extensions *in vitro* (unpublished observations) that could counteract the loss of oligodendrocytes following injury. (2) NX210 could directly stimulate myelination through the activation of its β1 integrin receptor, which is involved in the process of myelination [64].

For the first time, we are showing evidence that a SCO-spondin derived peptide, NX210, previously demonstrated to induce neuritogenesis [17], [19] is neuroprotective against oxidative stress-induced cell death, while promoting functional recovery in a rat model of SCI. These properties of NX210 justify further investigations to promote it as a candidate molecule in the medical management of patients with spinal cord injury. In addition, NX210 is a water soluble peptide which pharmacokinetic properties enable its administration into the lesion or into the subarachnoid space.
